# Modeling the Human Brain With *ex vivo* Slices and *in vitro* Organoids for Translational Neuroscience

**DOI:** 10.3389/fnins.2022.838594

**Published:** 2022-02-24

**Authors:** Giovanna O. Nogueira, Patricia P. Garcez, Cedric Bardy, Mark O. Cunningham, Adriano Sebollela

**Affiliations:** ^1^Department of Biochemistry and Immunology, Ribeirão Preto Medical School, University of São Paulo, São Paulo, Brazil; ^2^Institute of Biomedical Sciences, Federal University of Rio de Janeiro, Rio de Janeiro, Brazil; ^3^Laboratory for Human Neurophysiology and Genetics, South Australian Health and Medical Research Institute (SAHMRI), Adelaide, SA, Australia; ^4^Flinders Health and Medical Research Institute, Flinders University, Adelaide, SA, Australia; ^5^Discipline of Physiology, School of Medicine, Trinity College Dublin, Dublin 2, Ireland

**Keywords:** human, tissue, CNS, cell reprogramming, slices, disease models

## Introduction

Advances in translational neuroscience require experimental models recapitulating the complexity of the human brain. In contrast to conventional 2D cell culture models and animal models, 3D cultures have emerged as auspicious models to recapitulate the human brain structure and function in the laboratory setting (Jorfi et al., 2018). Human-derived 3D brain cultures are likely to recapitulate important human brain features in health and disease better than rodent brains (Humpel, 2015; Paşca, 2018), thus offering an optimal platform to examine pathophysiological mechanisms of diseases and demonstrate proof-of-concept evidence of the efficacy of new discoveries or treatments. As such, human-derived 3D cultures are perfectly positioned to play a critical role in experimental medicine.

Human brain tissue can be obtained from biopsies or through reprogramming technologies, including induced pluripotent stem cells. Recent advances in lab-made brain organoid technologies offer exciting opportunities for learning about neurological and psychiatric disease mechanisms (Quadrato et al., 2016). In addition, access to live brain tissue from patients, with minimal ethical concern, expand prospects for pre-clinical validation of therapeutics using human brain slice cultures (Martinez et al., 2011; Jones et al., 2016; Parker et al., 2017; Horowitz et al., 2020; Almeida et al., 2021). Despite these exciting advances, important drawbacks still limit the use of human-derived organoids and slice cultures. Brain organoids remain artificial and would benefit from more rigorous comparison with human brain tissue directly obtained from biopsies. In this article, we identify limitations and highlight opportunities for expanding the potential contribution of cutting-edge brain organoid technologies and more classical organotypic slices of adult human brain biopsies for translational neuroscience.

## Brain Organoids: The Beginning of a New Age

**Figure 1:**
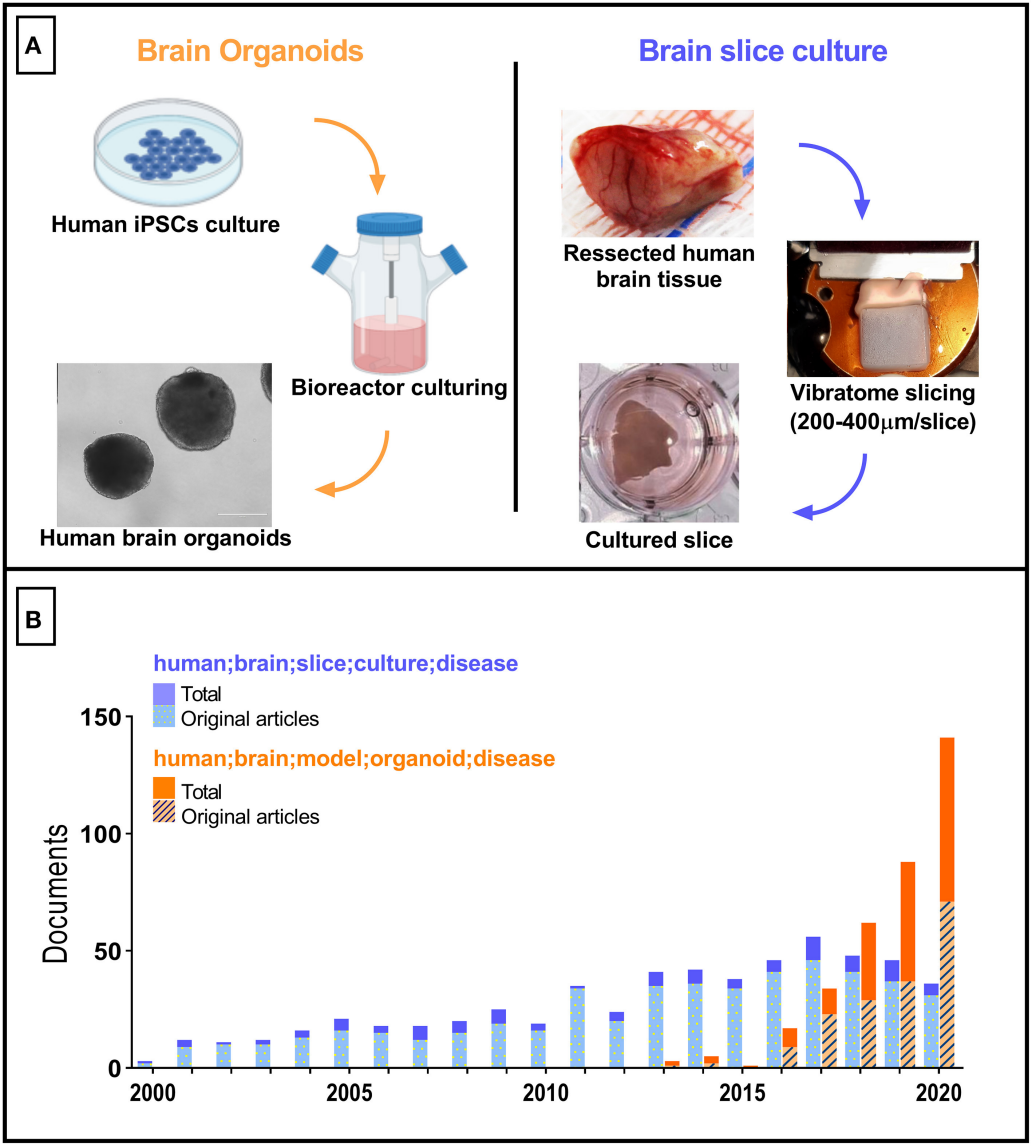
Tridimensional human brain-derived models comparative. **(A)** Summarized schematics of human brain organoids (Created with BioRender.com) and slice cultures preparation processes. **(B)** Number of research articles per year between 2000 and 2020. Blue bars correspond to the documents retrieved with “*human; brain; slice; culture; disease*” as search terms. The total number of documents was 591 (first document retrieved published in 1987). Orange bars correspond to the documents retrieved with “*human; brain; model; organoid; disease*” as search terms. Total number of documents equals 359 (one excluded due to retraction). The Scopus database was used.

The so-called brain organoids have gained prominence to become one of the most utilized *in vitro* nerve cells culture platforms in recent years. Brain organoids (Figure 1A, left) are 3D structures derived either from embryonic stem cells (ESCs) or induced pluripotent stem cells (iPSCs). Since its first description (Eiraku et al., 2008), brain organoids have been used to elucidate molecular alterations in human brain neurodevelopment associated with disorders such as autism (Mariani et al., 2015) and microcephaly (Lancaster et al., 2013; Camp et al., 2015; Garcez et al., 2016). In those investigations, brain organoids were thought to fill the gaps observed in typical rodent CNS-derived models. These shortcomings include significant differences in gene expression, protein sequences, proliferative zone composition, and others, in comparison to humans (Hodge et al., 2019).

In the afore-mentioned studies, brain organoids were chosen due to their capacity to reproduce early stages of brain development, allowing the investigation of disease onset in neurodevelopmental disorders (Tambalo and Lodato, 2020). Conversely, the production and maintenance of organoids that reproduce features of the mature human brain is still a major challenge. A few recent studies have reported the use of human brain organoids to investigate molecular mechanisms underlying neurodegenerative diseases typical of the aged human brain, such as Alzheimer's and Parkinson's diseases. Raja et al. (2016) applied iPSCs derived from Alzheimer's Disease (AD) patients to develop brain organoids. Interestingly, these patient-derived organoids recapitulated some of the key molecular hallmarks of AD, such as amyloid aggregation and tau protein hyperphosphorylation. In another study, Lin et al. (2018) investigated APOE4 expression in different brain cell types using patient-derived iPSC with APOE4 allele inserted by CRISPR/Cas9 technology. The use of this model allowed the authors to dissect the contribution of this gene variant, a well-known risk factor for AD, at cellular and molecular levels. Increased secretion of Aβ_42_ by neurons impaired lipid metabolism in astrocytes and pro-inflammatory microglial morphology. Also of note is the work by Kim et al. (2019), which used midbrain organoids generated from human iPSCs to examine pathogenic mechanisms associated with a gene mutation suspected to be linked to the onset of sporadic Parkinson's Disease (PD). Collectively, these studies position the use of patient-derived iPSCs as a promising approach to unravel mechanisms of neurodegenerative diseases such as AD and PD. However, the actual degree of similarity between human iPSC-derived brain organoids and the adult human brain have not yet been carefully assessed and require further investigation.

Despite the advances that have been made with respect to brain organoids development and their applications, several issues still hamper the acceptance of this approach as a model of the mature human brain. Examples include incomplete cortical lamination, lack of extracellular matrix cues (e.g. reelin and microglia-secreted metalloproteinases), mitochondrial dysfunction, and the reversal of genomic/mitochondrial DNA age-associated damage by the re-programming process (Franco and Müller, 2011; Mahmoudi and Brunet, 2012; Gerakis and Hetz, 2019; Crapser et al., 2021; Yadav et al., 2021). In this regard, adult human brain-derived slice cultures, which may be prepared from either tissue collected from brain surgeries or *post-mortem* tissue (reviewed in Qi et al., 2019) still appear as an optimal model from this perspective. Surprisingly, the number of articles reporting the use of slice cultures from human brains has been essentially constant over the past decade, with a tendency to decrease since the beginning of the brain organoids age (Figure 1B). Although this may be in part explained by regulatory and ethical constraints, and by the need to create an efficient workflow connecting hospital surgical rooms to tissue culture facilities in basic science laboratories, we advocate that these bottlenecks must be overcome to fuel an advance in our capacity to model diseases of the adult human brain.

## Brain Slice Cultures: A Powerful but Neglected Model

Brain slice cultures (Figure 1A, right) are particularly useful in the evaluation of mature brain features in health and disease. After its first description using rat brain tissue (Gähwiler, 1981), and the development of *postmortem* human brain culturing protocols (Verwer et al., 2002), different brain slice cultures protocols have been reported allowing either short (up to 10 days *in vitro*) or long-term (30 days or more) viability of live human brain tissue. This *ex vivo* model significantly preserves the cytoarchitecture, cellular diversity and extracellular matrix composition, spatial distribution, and connections between nearby cells and short distance circuits - although global network remodeling and cells activation in response to slicing have been reported (Fernandes et al., 2019; Qi et al., 2019; Schwarz et al., 2019). The presence of microglial and other non-neuronal cells (astrocytes and oligodendrocytes) allows a life-stage sensitive evaluation of brain responses, including the aged brain (Barth et al., 2021). Brain slice cultures also provide superior preservation of anatomical and connectivity differences between brain regions, in comparison to spherical organoids (Croft et al., 2019).

Brain slice cultures have been used as a platform to study morphological, biochemical, and functional responses of human CNS to toxic stimuli. For instance, human brain slices have been used to study injury response, and results pointed to microglia and astrocyte morphological alterations following *in vitro* severe injury (Verwer et al., 2015). Furthermore, glioblastoma progression in human brain slice cultures was successfully followed using electrophysiological recordings and changes in gene expression profiles through RNA-seq analysis (Ravi et al., 2019). In an attempt to study AD-associated amyloid-β oligomers toxicity in a relevant human brain model, brain slice cultures from adults were employed to map the effects of Aβ oligomers on global gene expression (Sebollela et al., 2012) and Tau phosphorylation (Mendes et al., 2018). The use of brain slice cultures has also been utilized in the epilepsy research (Eugène et al., 2014; Jones et al., 2016). Also of note, the feasibility of genetic manipulation in human brain slice cultures using HSV-1 viral vectors has been demonstrated (Ting et al., 2018); and human brain slice cultures have been useful in providing timescales that permit transduction of transgenes aimed to allow advanced imaging (GCaMP&FRET based probes; Le Duigou et al., 2018) or optogenetic manipulation (Andersson et al., 2016).

Interestingly, attempts have been made to produce slices from brain organoids. Giandomenico et al. (2019) and Qian et al. (2020) proposed the slicing methodology as an alternative to whole organoid cultures to improve survival *in vitro* by reducing hypoxia and allowing better nutrient delivery to cells. This strategy facilitates the use of brain organoids for a longer period in culture, providing a potential new method to model more mature brain stages. However, additional studies are required before such an approach can be validated as an adequate model for adult human acute brain slice studies.

## Discussion

Although human brain organoids and *ex vivo* slices efficiently recapitulate features of the human brain in health and disease, including some not observed in rodent brains or 2D cultures, there are still important limitations that need to be addressed before these human-derived 3D cultures become widespread employed models at their full potential. While brain organoids are not yet capable of resembling mature brains, and still a high-cost method; brain biopsies rely on a close collaboration between basic researchers and clinicians (Jones et al., 2016), making it difficult to be readily adopted in many research centers. Other important aspects to be considered when using human brain slice cultures is the molecular and cellular status of the tissue sample used to prepare each culture, since these are surrounding tissue from epileptic foci or brain tumors, and therefore may present alterations compared to a non-diseased brain (Johnson et al., 2015; Miller-Delaney et al., 2015), in addition to acute injury stress driven by the slicing process. In contrast, the organoid approach is advantageous in that the iPSCs can readily be obtained from both diseased and control donors, thus allowing a side-by-side comparison between healthy and diseased brain tissue, as in studies that mapped genetic predispositions in AD and PD (Israel et al., 2012; Tran et al., 2020). We believe those limitations can be overcome by exploring the potential of both models in a complimentary fashion.

Human brain slice cultures present an important limitation of tissue availability. However, they can be seen as a gold standard in terms of cell diversity, neuronal and cell-cell connectivity, and response to neuroactive compounds (Schwarz et al., 2019; Barth et al., 2021), allowing its establishment as a reference to optimize protocols to produce organoids that efficiently model the mature human brain. In this sense, adult human brain slices constitute a preferential tool to validate brain organoids molecular and functional responses to a range of stimuli (drug treatments, neurotoxins, infection, gene expression manipulations), in addition to enable the identification of aging markers to be used as references for *in vitro* aging protocols of brain organoids. As an interesting initiative in this regard, differential protein expression between fetal human brain and brain organoids has been determined using shotgun proteomics (Nascimento et al., 2019). By advancing in approaches like this, adult human brain cultures could be explored as a steppingstone for more accurate disease modeling using brain organoids, with the bonus of providing new insights into human central nervous system functioning.

The increasing development of 3D models based on stem cells reprogramming clearly defines the pathway taken by the research community towards the development of organoids as the main tool to reproduce complex biological systems in the laboratory. Importantly, this pathway is aligned with a global effort to reduce the number of animals in research along with the need to overcome the gap left by animal research in translational science through the replacement of animal models by human-derived experimental platforms (Prescott and Lidster, 2017). Choosing the most adequate experimental model depends on its proximity to the target system, and on how established are the methods to prepare it. Despite the limitations and drawbacks of brain organoids, their impact and importance are clearly bigger, and with improved protocols, it seems to be the future of human cellular neuroscience research.

Nevertheless, while the number of studies using brain organoids increases rapidly, the number of articles using human brain slice culture trends towards a reduction (Figure 1B). This in part could be explained by the novelty of brain organoids, resulting in a preference by scientists to adopt a cutting-edge technology. In the case of human brain slices, only ten research groups seem to be responsible for the majority of published articles using this model (when using as threshold publication of at least three articles as corresponding author, and the keywords “*human; brain; slice; culture; disease*”). Since the preparation of human brain slices and organotypic culture techniques are well described, opening spaces in top-tier journals dedicated to the communication of studies reporting this model could stimulate scientists and surgeons to work together and consequently increase the publication rate of translational studies. This action could fuel the conversion of brain slice cultures from a marginally explored tool to a leading approach in translational neuroscience. Brain organoids are still far from reproducing in exquisite detail several anatomical, physiological, and molecular aspects of the human brain across every life stage. Thus, human brain slice cultures could serve as a critical reference technique along the process of developing and validating brain organoids directed to different life stages.

## Author Contributions

GN and AS contributed to drafting the original manuscript. PG, CB, and MC critically revised the manuscript and contributed to the writing process. All authors contributed to the manuscript and agree with its content.

## Funding

This work was supported by FAPESP (Grants: #2014/25681-3), FAEPA, CNPq, and CAPES.

## Conflict of Interest

The authors declare that the research was conducted in the absence of any commercial or financial relationships that could be construed as a potential conflict of interest.

## Publisher's Note

All claims expressed in this article are solely those of the authors and do not necessarily represent those of their affiliated organizations, or those of the publisher, the editors and the reviewers. Any product that may be evaluated in this article, or claim that may be made by its manufacturer, is not guaranteed or endorsed by the publisher.
